# Exploration of different statistical approaches in the comparison of dopamine and norepinephrine in the treatment of shock: SOAP II

**DOI:** 10.1186/s13054-024-05016-9

**Published:** 2024-09-10

**Authors:** Fernando G. Zampieri, Sean M. Bagshaw, Hassane Njimi, Jean-Louis Vincent, Daniel DeBacker, SOAP II Investigators, P. Biston, J. Devriendt, C. Madl, D. Chochrad, C. Aldecoa, A. Brasseur, P. Defrance, P. Gottignies, R. Kitzberger, U. Holzinger, A. Roman, D. De Bels, S. Anane, S. Brimioulle, M. Van Nuffelen, M. VanCutsem, J. Rico, J. I. Gomez Herreras, C. Mélot

**Affiliations:** 1 Department of Critical Care Medicine, Faculty of Medicine and Dentistry, University of Alberta, and Alberta Health Services, Edmonton, Canada; 2 Department of Intensive Care, Erasme University Hospital, Université Libre de Bruxelles, Brussels, Belgium; 3 Department of Intensive Care, CHIREC Hospitals, Université Libre de Bruxelles, Brussels, Belgium

**Keywords:** Dopamine, Norepinephrine, Shock, Critical care, Randomized, Bayesian

## Abstract

**Background:**

Exploring clinical trial data using alternative methods may enhance original study’s findings and provide new insights. The SOAP II trial has been published more than 10 years ago; but there is still some speculation that some patients may benefit from dopamine administration for shock management. We aimed to reanalyse the trial under different approaches and evaluate for heterogeneity in treatment effect (HTE).

**Methods:**

All patients enrolled in SOAP II were eligible for reanalysis. We used a variety of methods including the win-ratio (WR), a Bayesian reanalysis stratified according to shock type, and both a risk-based and effect-based explorations for HTE. The methods were applied to different endpoints, including a hierarchy of death, new use of renal-replacement therapy (RRT), and new-onset arrhythmia; 28-day mortality; a composite endpoint (mortality, new use of RRT, and new-onset arrhythmia), and days alive and free of ICU at 28-days (DAFICU28).

**Results:**

A total of 1679 patients were included (average age was 64.9 years, 57% male, 62% with septic and 17% with cardiogenic shock). All analysis favoured norepinephrine over dopamine. Under the WR approach, dopamine had fewer wins compared to norepinephrine (WR 0.79; 95% confidence intervals [CI] 0.68–0.92; *p* = 0.003), evident in both cardiogenic and septic shock subgroups. The Bayesian reanalysis for type of shock showed, for dopamine, a probability of harm of 0.95 for mortality, > 0.99 probability of harm for composite endpoint, and 0.91 probability of harm for DAFICU28. The fewer DAFICU28 with dopamine was more apparent in those with cardiogenic shock (0.92). Under the risk-based HTE, there was a high probability that dopamine resulted fewer DAFICU28 in the highest quartile of predicted mortality risk. The effect-based HTE assessment model did not recommended dopamine over norepinephrine for any combination of possible modifiers including age, type of shock, presence of cardiomyopathy, and SOFA score. Receiving dopamine when the effect-based model recommended norepinephrine was associated with an absolute increase in composite endpoint of 6%.

**Conclusion:**

The harm associated with the use of dopamine for the management of shock appears to be present in both septic and cardiogenic shock patients. There was no suggestion of any subgroup in which dopamine was found to be favourable over norepinephrine.

**Supplementary Information:**

The online version contains supplementary material available at 10.1186/s13054-024-05016-9.

## Background

Choice of vasopressor is a crucial issue in patients with shock admitted to intensive care units (ICU). Dopamine was commonly used as first line vasopressor in patients with shock; however, norepinephrine has been favoured over the last couple decades after trials suggesting that norepinephrine could be associated with improved outcomes and fewer adverse effects [1, 2]. The landmark trial comparing norepinephrine and dopamine is the Comparison of Dopamine and Norepinephrine in the Treatment of Shock trial (SOAP II) which enrolled 1679 critically ill patients with shock to either norepinephrine or dopamine. SOAP II reported neutral results for the primary outcome of 28-day mortality (odds ratio [OR] with dopamine, 1.17; 95% confidence interval [CI] 0.97–1.42; *p* = 0.10) [3]. However, the trial found important differences, including more adverse events with dopamine [3].

Interpreting neutral clinical trials that include a heterogeneous population is challenging. On one hand, the effect size of SOAP II points towards possible harm with the use of dopamine; and a secondary subgroup analysis suggested that harm could be driven mostly by patients with cardiogenic shock, but the *p*-value for interaction was 0.12, making a strong statement not completely compatible under the frequentist framework. In addition, physicians continue to use dopamine as a vasopressor agent, suggesting that it may still be considered suitable in some conditions. There is growing interest for different statistical methods that can either provide hierarchical assessment of composite endpoints (such as the win ratio [WR]) [4], probabilistic statements on the results (mostly through Bayesian methods [5]) and exploring heterogeneity in treatment effects (HTE), which may provide additional information from randomized trials [6].

We therefore reanalyzed the SOAP II trial using different statistical methods, including a hierarchical assessment of composite endpoints using WR [4], Bayesian reanalysis accounting for interaction of shock type and intervention [5], probabilistic methods of HTE based on risk [6] and effect-based approaches [7]. We hypothesized that these methods would provide additional insights that would both serve to showcase novel methods of analysis for randomized trials and augment the findings from the original SOAP II results.

## Methods

### Patients

All patients included in the SOAP II trial (raw original dataset).

### SOAP II overview

SOAP II was a multicenter, randomized, clinical trial, where patients were randomly allocated to receive either dopamine or norepinephrine as the first-line vasopressor therapy to manage shock. If the blood pressure could not be maintained with a dose of 20 μg per kilogram of body weight per minute for dopamine or a dose of 0.19 μg per kilogram per minute for norepinephrine, open-label norepinephrine, epinephrine, or vasopressin could be added. The primary endpoint was all-cause 28-day mortality. Fully anonymized data provided by SOAP II investigators was used for this analysis.

### Missing data handling

Continuous or factor variables with missing values up to 1% of the sample size were imputed using median or most common level.

### Endpoints and rationale

For the WR approach, two hierarchical approaches were used: (1) 28-day mortality, use of renal replacement therapy (RRT), and occurrence of new arrhythmia was used, and (2) 28-day mortality and ICU length-of-stay (LOS). Those endpoints encompass relevant hierarchies that could have been chosen in SOAP II; both use mortality as first endpoint (to avoid competing risks). The difference is how ties in mortality are dealt with. The first hierarchy adds two additional layers including use of RRT and arrhythmias, which could be affected by dopamine use. The second hierarchy, a simpler combination of mortality and LOS, is conceptually similar to days alive and free of ICU endpoints. There was no stopping due to early ties. WR above 1 would suggest dopamine is better (i.e., results in more wins). For the Bayesian reanalysis according to type of shock and HTE explorations, three different endpoints were used: 28-day mortality; a composite of 28-day mortality, new use of RRT and occurrence of new arrhythmia (hence using the first WR hierarchy in a single composite endpoint); and days alive and free of ICU at 28 days (DAFICU28; which conceptually approaches the second win ratio hierarchy used). Patients that died within 28 days received zero DAFICU28.

### Statistical analysis

The analysis method depended on the approach used to explore SOAP II:

#### WR analysis

We calculated the WR for dopamine over norepinephrine using both hierarchical endpoints stratified according to shock type (grouped in cardiogenic, septic, and other; the “other” category used for analysis includes all types of shock that are not cardiogenic and septic; detailed explanation is provided in Table 1 legend) [8]. Results are presented as a WR with 95% confidence interval (CI), and *p* values. Values of WR below 1 suggest a harm for dopamine in the composite endpoint (i.e., fewer wins).

**Table 1:** Patient features according to randomization arm

Characteristic	Dopamine N = 858	Norepinephrine N = 821
Admission		
Age, mean (SD)	65 (15)	65 (14)
Sex, n (%)		
Female	351 (41%)	372 (45%)
Male	507 (59%)	449 (55%)
APACHE II, median (IQR)	21 (15, 28)	20 (15, 27)
Cardiomyopathy, n (%)*		
No	432 (50%)	399 (49%)
Yes	426 (50%)	422 (51%)
Type of shock, n (%)		
Anaphylactic	3 (0.3%)	4 (0.5%)
Cardiogenic	135 (16%)	145 (18%)
Hypovolemic	138 (16%)	125 (15%)
Other†	40 (4.7%)	45 (5.5%)
Septic	542 (63%)	502 (61%)
Mechanical Ventilation, n (%)	615 (72%)	580 (71%)
Renal replacement therapy, n (%)	63 (7%)	61 (7%)
SOFA, points, mean (SD)	9.0 (3.8)	8.6 (3.8)
Respiratory	2.50 (1.27)	2.27 (1.27)
Cardiovascular	3.26 (1.01)	3.16 (1.05)
Coagulation	0.57 (1.00)	0.55 (0.95)
Hepatic	0.39 (0.89)	0.38 (0.86)
Neurologic	0.96 (1.46)	0.89 (1.40)
Renal	1.32 (1.55)	1.32 (1.58)
Outcomes		
New use of renal replacement therapy, n (%)	64 (7%)	81 (10%)
Arrhythmia	207 (24%)	102 (12%)
Days Alive and Free of ICU, mean (SD)	8 (11)	9 (11)
28-day mortality	450 (52%)	398 (48%)

*Cardiomyopathy as defined by enrolling site. † includes other types of shock that did not fit on other categories and can include obstructive or unspecified shock in the opinion of the enrolling site. For analyses, shock types were categorized in “septic”, “cardiogenic”, and “other”, which includes “other”, “anaphylactic”, and “hypovolemic” types of shock

#### Bayesian reanalysis of the main trial accounting for the interaction of the intervention with shock type

The effect of dopamine versus norepinephrine for 28-days mortality was assessed using a Bayesian regression model for randomization arm on outcome adjusted for type of shock and the interaction between type of shock and intervention. A neutral prior was used for the effect of the intervention (neutral prior for log odds ratio with mean zero and standard deviation of 0.355 [5]). Relevance of the interaction was estimated according for the Bayes factor (BF) for a model with and without interaction, with a BF above 10 being supportive of presence of interaction (i.e., the model with interaction is at least 10 times more likely to fit the data than the model without interaction). If the BF was above 10, we planned to explore the effects of the intervention on each shock type; otherwise, we planned to report only the result of the model without interaction, including the odds ratio and absolute risk differences for mortality with dopamine when compared to norepinephrine together with its probability of direction and its 95% high density intervals (HDI). A region of practical equivalence (ROPE) was defined as differences in absolute risks of 0.01. A probability of direction above 0.90 was suggestive of an effect; a probability of direction above 0.95 with less than 0.05 of the posterior mass of absolute risk difference within the ROPE (%ROPE < 0.05) was considered as strong evidence of effect. A similar approach was applied for the composite endpoint of death, new use of RRT or new arrhythmia. For DAFICU28, a Bayesian linear model was used with; cut off for probability of direction as well as BF criteria were similar; ROPE was defined as a difference of plus or minus one day for DAFICU28.

#### Risk-based heterogeneity in treatment effect

This approach followed the PATH statement [6]. We first performed a first level customization of APACHE II in the trial population and assessed its discrimination capability (through receiver-operating characteristics curve) and calibration (using calibration belts) [9]. Customization was necessary due to the lack of crude data on reason for admission allowing original APACHE II predictions to be calculated. We then generated quartiles of predicted risk of death based on customized APACHE II [10] (cAPACHE II) predicted probabilities. The prediction risks quartiles were then used for a Bayesian logistic regression with 28-day mortality as outcome together with study randomization arm and their interaction. Presence of risk-based HTE was defined as a BF above 10 for the model with versus a model without the interaction for cAPACHE II quartiles for the primary outcome. These risk categories from APACHE II were also used for risk-based HTE explorations for composite endpoint and DAFICU28. Results are presented as odds ratios, absolute risk differences, or mean differences, according to enrollment arm with their 95% HDI. Same rules for stating association and ROPE were used.

#### Effect-based heterogeneity in treatment effects

This analysis was designed to apply the S-learner technique using SOAP II for the composite endpoint [7]. In brief, the S-learner is a technique where a prediction model allowing interaction between intervention arm and key covariates is trained in part of the original dataset (“train set”; defined as a 0.60 of total SOAP II sample size) [7]. The model was adjusted for age, type of shock, presence of cardiomyopathy at baseline, and sequential organ failure assessment (SOFA [11]) score, all interacting with intervention arm. The model was then used to generate two counterfactual predictions in remaining sample (“test” set); counterfactuals were generating by first changing all patients to dopamine arm, obtaining predictions, then changing all patients to norepinephrine arm and obtaining a second set of predictions. The difference between predictions in the test set are an estimate of individualized treatment effects (ITE) for those patients. The model would then recommend one specific arm if the probability of benefit for that arm in that specific patient was above 0.90; if the differences in expected predicted outcomes did not reach the threshold, the model would not make a recommendation. If a recommendation could be made for at least 25% of the test set, the association between model recommendations and outcome would be assessed using a Bayesian logistic regression model with interaction between recommendation and study arm. We reported outcome differences according to recommended versus received treatment in an exploratory fashion following the same principles as the other Bayesian models.

#### Additional exploratory analyses

Additional exploratory analysis included assessment on whether heart rate would be an effect modifier for dopamine use; this included direct interaction analyses and an analysis also adjusted by type of shock.

All analysis were performed in R version 4.3.2 with packages {BuyseTest} [8], {brms} [12], and {marginaleffects} [13].

## Results

A total of 1679 patients were included. Patient features are shown in Table 1. Average age was 64.9 years (standard deviation—SD—14.6) and most patients were male (57%). Septic shock was the most common etiology for shock (1044 patients—62%). Mechanical ventilation was used at enrollment in 1195 patients (71.2%). A summary of methods and results is shown in Table 2.

**Table 2:** Summary of analyses, results, and interpretations discussed in the manuscript

Analysis	Endpoint	Motivation	Result	Interpretation
Win ratio	Hierarchy of death → RRT → arrhythmia	Combine mortality with two outcomes that were previously suggested with dopamine use (namely possible renal protective effects and possible higher arrhythmia occurrence	Less wins for dopamine (WR = 0.79 (95% CI 0.68–0.92; *p* = 0.003) for untied comparisons. Results consistent for cardiogenic and septic shock groups	Dopamine was associated with less wins than norepinephrine suggesting, under this approach, that it was inferior to norepinephrine. Results of WR, however, are not easily translatable to clinicians
	Hierarchy of death → ICU LOS)	Combine mortality and ICU LOS in a single endpoint	Neutral results (WR = 0.96; 95% CI 0.86–1.08; *p* = 0.51)	Using this hierarchy under a WR approach, results were neutral hence corroborating with the idea that arrhythmia was the larger driver factor in the first hierarchy approach
Bayesian reanalysis for shock type	28-day mortality	Reassess the main trial while adjusting for type of shock an interaction	No clear interaction for shock type and type of vasopressor; however, there was a high probability of harm with dopamine (0.95), with a median increase in mortality of 0.04 (95% HDI from − 0.01 to 0.09)	A simple Bayesian reanalysis under a neutral prior highlight that there was a high probability of harm with dopamine
	Composite endpoint	Reassess the main trial using a composite endpoint of death, RRT, and arrhythmia while adjusting for type of shock an interaction	No clear interaction for shock type and type of vasopressor; however, there was a very high probability of harm with dopamine (0.99), with a median increase in endpoint of 0.09 (95% HDI from 0.04 to 0.12)	Very high probability that dopamine was harmful was harmful, which should suffice by itself as evidence against the drug
	DAFICU28	Reassess the main trial using a more granular endpoint that has become common over last decade while adjusting for type of shock an interaction	There was a suggestion of association between type of shock and DAFICU, with the highest probability on cardiogenic shock (0.92). Overall, dopamine was associated with − 0.7 DAFICU28 (95% HDI − 1.58 to 0.92 days)	There was a suggestion for the interaction between vasopressor type and DAFICU28, but overall signal for harm is sustained
Risk-based HTE assessment	28-day mortality	Assess risk-based HTE using customized APACHE II predictions for 28-day mortality	No clear interaction between predicted APACHE II quartiles and outcomes. Results suggesting harm of dopamine similar to analysis stratified by type of shock	No clear risk-based HTE for primary endpoint for dopamine versus dopamine
	Composite endpoint	Assess risk-based HTE using customized APACHE II predictions for composite endpoint	No clear interaction between predicted APACHE II quartiles and outcomes. Results suggesting harm of dopamine similar to analysis stratified by type of shock	No clear risk-based HTE for composite endpoint for dopamine versus norepinephrine
	DAFICU28	Assess risk-based HTE using customized APACHE II predictions for DAFICU	Risk-based HTE was probable, with a suggestion of greatest harm for dopamine in the highest quartile of APACHE II predictions	Using a more granular endpoint, some suggestion of risk-based HTE can be found in SOAP II, with increasing harm for dopamine as illness severity increased
Effect-based HTE assessment	Composite endpoint	Use a causal HTE model adjusted for key covariates to try to obtain more information regarding possible THE	A model recommendation recommended norepinephrine for most patients and did not recommend dopamine	A simple S-learner recommended norepinephrine for most patients in the test set; no specific combination of possible mediators resulted in a recommendation favouring dopamine

RRT, Renal replacement therapy; DAFICU28, Days alive and free of ICU at 28 days; WR, Win Ratio; THE, Heterogeneity in treatment effects

### WR results

**Fig. 1:**
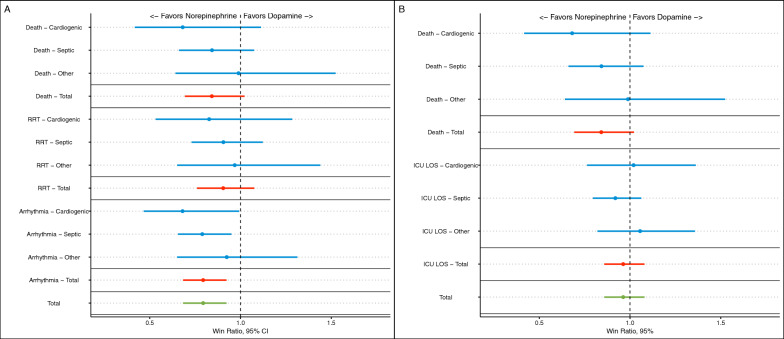
Results for the win ration (WR) analysis for the first hierarchical approach (**A** and **B**). **A** Stratified win ratio results for the first hierarchical approach (mortality, use of renal replacement therapy—RRT, and occurrence of arrhythmia). **B** Stratified win ratio results for the second hierarchical approach (mortality and ICU length-of-stay—LOS)

A cross tabulation of the components of the first WR hierarchy is shown in eTable 1. The overall WR for dopamine versus norepinephrine for the first hierarchical approach was 0.79 (95% CI 0.68–0.92; *p* = 0.003), suggesting that dopamine resulted in less wins than norepinephrine. For the secondary hierarchy, WR was 0.96 (95% CI 0.86–1.08; *p* = 0.51). Results for each hierarchical level according to strata for both approaches are shown in Fig. 1 and eFigure 1.

### Bayesian reanalysis results for the interaction with shock type

For the standard Bayesian reanalysis, the BF for the primary endpoint for a model with interaction versus without interaction was 0.8, suggesting that it is unlikely that a significant interaction between enrolment arm and type of shock for this endpoint. There was a suggestion that dopamine was worse than norepinephrine (probability of harm of 0.95), with 0.09 of the posterior within the region of practical equivalence. The median increase in mortality with dopamine was 0.04; 95% HDI from − 0.01 to 0.09 (odds ratio of 1.18; 95% HDI from 0.96 to 1.39). Model syntax and diagnostics are shown in ESM (eFigure 2).

For the composite endpoint result, BF for model with interaction was 2.84, not supportive of relevance of the interaction between study arm and type of shock. There was strong association between dopamine and more composite endpoints than norepinephrine in this analysis (probability of harm > 0.99; %ROPE < 0.01; absolute risk difference of 0.08, 95% HDI 0.04–0.12; odds ratio of 1.44, 95% HDI from 1.18 to 1.73). Model syntax and diagnostics are shown in ESM (eFigure 3).

**Fig. 2:**
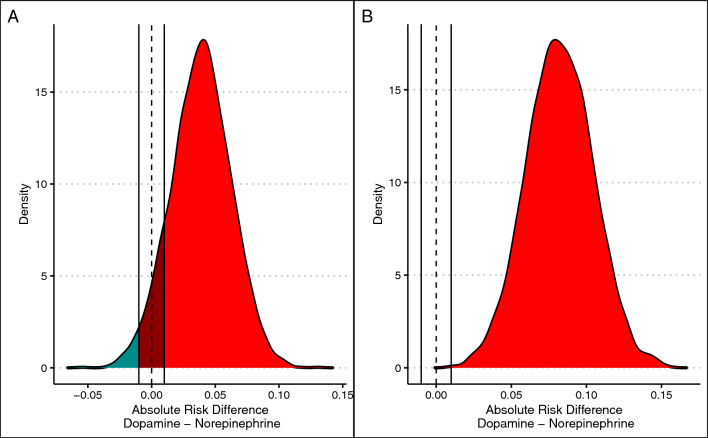
Bayesian reanalysis for 28-day mortality and composite endpoint. Blue areas represent benefit for dopamine, red areas represent harm for dopamine (benefit for norepinephrine), and dark red areas represent the region of practical equivalence. **A** Posterior distribution of adjusted risk difference for mortality. **B** Posterior distribution of adjusted risk difference for composite endpoint of mortality, use of renal replacement therapy, and mortality

**Fig. 3:**
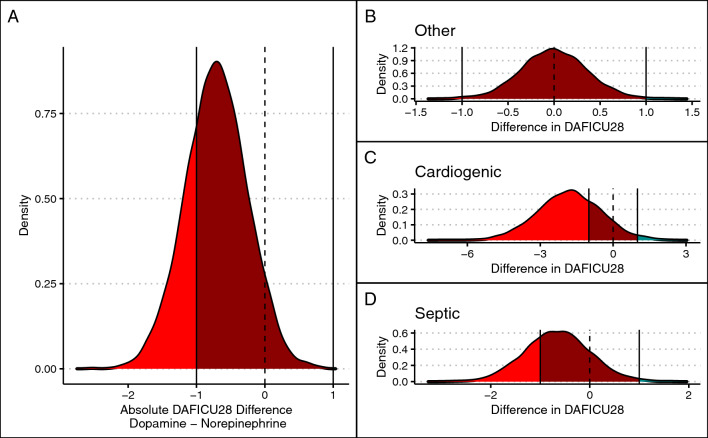
Bayesian reanalysis for day-alive and free of ICU at 28 days (DAFICU28). **A** Overall difference in DAFICU28 in a model adjusted for shock type. **B**, **C**, and **D** show the posterior distribution according to shock type. Areas in blue mark benefit for dopamine; areas in red mark harm for dopamine; region of practical equivalence—ROPE—is highlighted in dark red

For the DAFICU28 analysis, results were supportive of an interaction between enrollment arm and outcome (BF of 20). For all types of shock, there was an association between dopamine use and less DAFICU28 (mean difference of − 0.70 days; 95% HDI − 1.58 to 0.16 days; probability of harm of 0.91); these results varied according to shock type: probability of harm of dopamine was 0.50, 0.92, and 0.85 for patients with other types of shock, cardiogenic shock, and septic shock, respectively. Differences in DAFICU28 also varied according to shock type (0.00, 95% HDI from − 0.68 to 0.68; − 1.79, 95% HDI − 4.31 to 0.62; and − 0.65, 95% HDI − 1.92 to 0.56 for other types of shock, cardiogenic shock, and septic shock, respectively). Results for these analyses are shown in Fig. 2 (28-day mortality and composite endpoint) and Fig. 3 (DAFICU28). Model syntax and diagnostic plots are shown in the ESM (eFigure 4).

### Risk-based THE

**Fig. 4:**
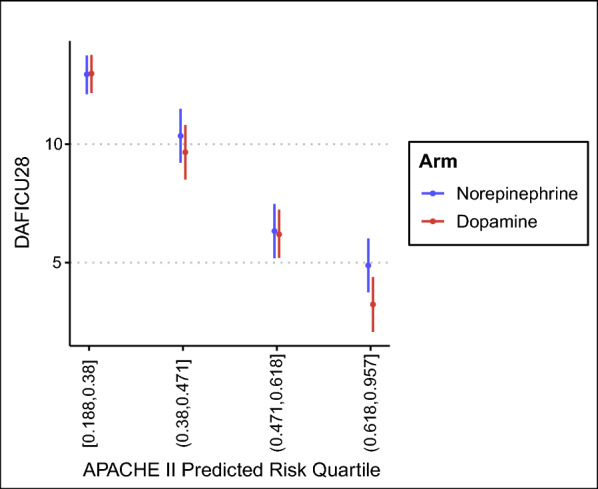
Risk-based HTE results for days alive and free of ICU at 28 days—DAFICU28—according to customized APACHE II predicted mortality and randomization arm. Note that the differences in DAFICU28 are more pronounced for more severe patients (last quartile). Numeric values are shown in Table 3. Parenthesis are used to denote open intervals and brackets are used to denote the interval is closed at that end

Discrimination and calibration of customized APACHE II is shown in eFigure 5. Model syntaxes and diagnostic plots are provided in the ESM (eFigure 6–8). There was no clear sign of presence of risk-based HTE based on quartiles of APACHE II for 28-day mortality or the composite endpoint (BF of 2.5 and 1.40, respectively). Since there was no strong evidence of interaction, no summaries according to quartiles were calculated for those endpoints. There was a strong suggestion for the interaction between DAFICU28 and quartiles of cAPACHE II predicted mortality (BF of 75), with a suggestion of harm for dopamine concentrated in the higher quartile of predicted mortality for cAPACHE II (mean difference of − 1.64 days; 95% HDI − 3.62 to 0.48; probability of harm of 0.95). Results are visually shown in Fig. 4 with numeric values in Table 3.

**Table 3:** Results for risk-based HTE analysis

	Odds ratio (95% HDI)	Absolute difference (95% HDI)	P(harm)	%ROPE*
28-day mortality	1.13 (0.94–1.38)	0.03 (− 0.01 to 0.07)	0.90	0.17
Composite endpoint	1.41 (1.14–1.71)	0.07 (0.03–0.12)	0.99	< 0.01
DAFICU28	–	− 0.57 (− 1.40 to 0.30)†	0.91	0.85
Low quartile [0.188,0.38]	–	0.04 (− 0.60 to 0.68)†	0.55	1.00
Medium–low quartile (0.38,0.471]	–	− 0.71 (− 2.58 to 1.32)†	0.76	0.60
Medium–high quartile (0.471,0.618]	–	− 0.12 (− 2.15 to 1.76)†	0.54	0.73
High quartile (0.618,0.957]	–	− 1.64 (− 3.62 to 0.48)†	0.95	0.24

Results for 28-day mortality and composite endpoint are not reported according to quartiles due to lack of clear signal for interaction; only results for days alive and free of ICU at 28 days—DAFICU28—is reported across quartiles. Values inside brackets represent the cAPACHE II prediction quartile range. *Region of practical equivalence—ROPE—defined as 1% differences for 28-day mortality and compositive endpoint and as 1 day difference for DAFICU28. †difference in DAFICU28

### Effect-based THE

**Fig. 5:**
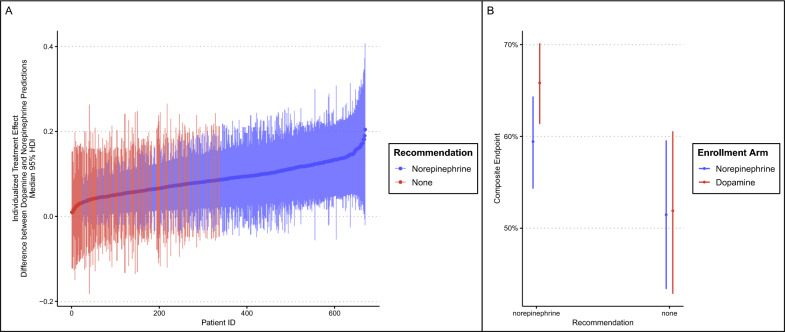
**A** “Individualized” (conditional) treatment effects (ITE) for patients in the test set according to the model adjusted for adjusted for age, type of shock, presence of cardiomyopathy at baseline, and SOFA score, all interacting with intervention arm. The differences between counterfactuals for each patient are displayed in (**A**), with patient identification sorted according to increasing ITE and y-axis showing the median difference and 95% HDI for the counterfactuals; a high value represents an increase in the counterfactuals between dopamine and norepinephrine, hence the higher the conceivable benefit for norepinephrine versus dopamine the higher the ITE. Note that the model could not make a recommendation in 27% of all patients. **B** Differences in outcome according to enrollment arm and suggestion obtained from the S-Learner. Receiving norepinephrine when the model recommended norepinephrine was associated with a decrease in composite endpoint; for patients for whom no recommendation was made, no clear differences between norepinephrine and dopamine were found for patients for whom no recommendation was made

Details of model syntax and technical aspects are shown in ESM (including eFigure 9 for model diagnostics). The results for individual prediction of ITE (differences in composite endpoint based on S-Learner) is shown in Fig. 5A. The S-learner could make a recommendation in 73% of the test set (489 out of 669 patients). The model recommended norepinephrine for 489 patients, could not make a recommendation in 180, and never recommended dopamine. A comparison between patients recommended norepinephrine versus those without recommendation is shown in eTable 2; patients recommended norepinephrine more frequently had septic shock (78%) and had higher overall SOFA score. Receiving dopamine when the model recommended norepinephrine was associated with a 0.06 increase in composite endpoint (95% HDI − 0.01 to 0.14, 0.95 probability of harm, %ROPE 0.06), visual representation in Fig. 5B. Receiving dopamine when no recommendation could be made was not clearly associated with changes in composite endpoint (median difference of 0.95% HDI from − 0.14 to 0.15).

### Additional analyses

Results were neutral for both the continuous interaction of heart rate and intervention arm (eFigure 10) and heart rate (divided in quartiles) study arm and type of shock (eFigure 11).

## Discussion

### Overall results

In this exploratory post-hoc analysis of the SOAP II trial using additional analytic strategies, we have expanded interpretation of the main trial findings. SOAP II reported neutral results for mortality but suggested that dopamine was associated with more adverse events, including arrhythmias [1]. The present analysis brings some additional important conclusions, some which are complementary to the original report and some that are confirmatory. From a confirmatory perspective, our results suggest that if mortality and arrhythmia are bundled in a composite endpoint, the harm associated with dopamine is clearly presented (as shown in the WR analysis using the first hierarchy and the Bayesian reanalysis using composite endpoint). From a complementary perspective, our results highlight that a simple Bayesian reanalysis adjusted for type of shock already suggested a high probability of harm for dopamine for 28-day mortality (0.95). If more granular endpoints were used, such as days alive and free of ICU at day 28, then an interaction between shock type and outcome became more apparent, with a suggestion of increased harm with use of dopamine in cardiogenic shock. The heterogeneity in treatment effect for dopamine versus norepinephrine was also evident in risk-based HTE assessment based on APACHE II scores, making it uncertain whether type of shock or simply overall baseline risk are the mediators of the effect. A simple effect-based model based on S-learner confirmed the results with the model suggesting use of norepinephrine only for over 70% of the test set and never recommending dopamine.

### Why is this analysis necessary now?

Revisiting trials through secondary exploratory analysis is a well-known phenomenon in critical care [14]. Traditionally, secondary analysis of a trial is done using specific subgroups of interest, which are prone to low power and magnification errors for positive results [15]. More recently, trials have started being reanalyzed using different endpoints and more advanced analytic methods, including Bayesian statistics or hierarchical endpoints [16, 17], often providing added insights beyond the original trial [16], but also generating concerns that excessive reanalysis and explorations will lead to spurious results or could be used to flip the primary conclusions of a clinical trial, specifically regarding the case of Bayesian analyses [18]. Dopamine is no longer recommended as first-line vasopressor in adults with septic shock [19] mostly due to SOAP II and the Patel et al. trials [1, 3, 20]. However, several guidelines for management of acute heart failure still mention dopamine use as a conceivable alternative mostly due to lack of supporting data against its use [21, 22]. In the recent DanGer trial, close to 25% of all patients with cardiogenic shock used dopamine [23]. Reappraising the SOAP II trial therefore represents an opportunity to both confirm the results for septic shock and provide further evidence for dopamine use in other types of shock, which are still lacking. This analysis therefore was needed to both confirm SOAP II results in the light of newer methods and to provide further evidence for other types of shock.

### What those analyses add?

This reanalysis provides an in-depth exploration of SOAP II considering methods that were not disseminated at the time of the original trial’s publication. The results are consistent. All analyses favoured, to some extent, norepinephrine versus dopamine for shock. The findings were mostly inconclusive for patient without septic or cardiogenic shock (“other” types of shock), probably due to the low number of events in this less-severely ill population. Our results highlight that dopamine should not be a drug of choice for any shock type. This is particularly true for patients at higher risk of death (highest predicted APACHE II mortality) where dopamine was associated with fewer days alive and free of ICU. Some other important results include the high probability of harm for dopamine, also considering DAFICU28 as an endpoint, in septic and cardiogenic shock populations. The fact that a simple effect-based model using S-learner was unable to recommend dopamine regardless of the baseline combination of possible modifiers is also a strong argument for not using dopamine as first-line under most circumstances. Considering that it is very unlikely that new trials will test dopamine for shock given the overall context, this reanalysis provide further confirmatory evidence that extends from septic shock.

In addition, we hope that this reanalysis also reinforces that, when done properly, reanalysis can confirm and extend the main results of a trial in a concise way. The number of analyses is purposely high to showcase different approaches that could have been considered had the SOAP II trial been designed more recently.

### Limitations

There are several limitations for this analysis. First, this is a post-hoc analysis based on previously published data, and hence should be viewed as exploratory. Second, the SOAP II trial was published more than 10 years ago and therefore, these findings may be limited due to the inevitable changes in ICU practices. Third, the number of analyses included in this analysis is extensive. We aimed to present the results of these analyses as streamlined as possible and provided a summary table of all our findings. Forth, all endpoints are anchored to mortality which was numerically higher in dopamine group in the trial; therefore, analyses using composite endpoints are essentially increasing power due to high number of events; this, however, is common practice in many trials in critical care and other fields. Fifth, definitions of ROPE were arbitrary in the lack of clear consensus on how to define equivalence for endpoints in critical care.

## Conclusion

Dopamine may be associated with increased harm when used in patients with shock. This effect seems to be present in patients with either septic or cardiogenic shock. There was no suggestion of any subgroup in which dopamine should be favourable over norepinephrine.

## Supplementary Information

Supplementary Material 1.
