# Supplementary material for: Exploration of different statistical approaches in the comparison of dopamine and norepinephrine in the treatment of shock: SOAP II
Source: Crit Care. 2024 Sep 10;28:299. doi: 10.1186/s13054-024-05016-9 (PMC11385121; doi:10.1186/s13054-024-05016-9)

Exploration of Different Statistical Approaches in the Comparison of Dopamine and Norepinephrine in the Treatment of Shock – SOAP II

Electronic Supplementary Appendix

INDEX

Analysis	Page
**Additional Information for Win Ratio Analysis**	2
**Bayesian Analysis**	3-5
**APACHE II customization**	6
**Risk-based HTE**	7-9
**Effect-based HTE**	10-12
**Other Analysis**	13

1. Additional Information for Win Ratio Analysis

eTable 1 – Cross tabulation of events in the first hierarchical WR according to randomization arm

**Endpoint**			**Arm**	
**Death**	**New RRT**	**Arrhythmia**	**Norepinephrine**	**Dopamine**
No	No	No	348 (55%)	289 (45%)
No	No	Yes	42 (31%	93 (69%)
No	Yes	No	29 (62%)	18 (38%)
No	Yes	Yes	4 (33%)	8 (67%)
Yes	No	No	305 (49%)	315 (51%)
Yes	No	Yes	45 (32%)	97 (68%)
Yes	Yes	No	37 (56)	29 (44%)
Yes	Yes	Yes	11 (55)	9 (45%)

eFigure 1 - Number of pairs available for comparisons and wins, losses, and neutral comparisons among each endpoint, stratified according to show type for the first (A) and second (B) hierarchical approaches.

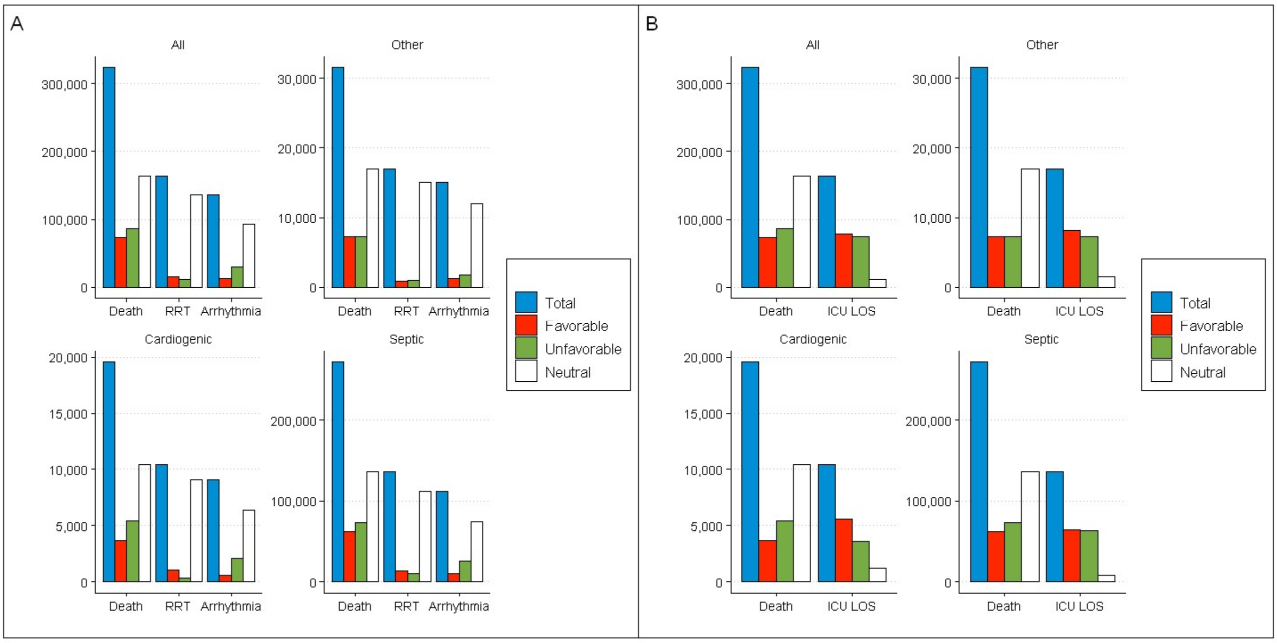

2. Bayesian Analysis

A similar syntax was used for most models for the Bayesian analysis. Model syntax and diagnostic plots are provided below.

Note that for all models Bayes Factors for model with and without interaction were done by creating a model with the interaction and one without it, and then comparing models using brms::bayes_factor(b1,b1ni), where "ni" refers to the model built without interaction.

Coefficients and contrasts were extracted using {marginaleffects}

a. Model Syntax for primary endpoint (28-day mortality): Note that the neutral prior was applied to intervention arm, but the remaining priors were kept as uninformative (flat). The prior concentrates 95% of its probability mass between odds ratio of 0.5 to 2.0.

```
myprior <- prior(normal(0,0.355), class="b", coef="arm1")
b1 <- brm(death ~ arm * type_of_shock, family="bernoulli", chains = 4, cores = 4, seed = 123, prior = myprior, data = df,save_pars = save_pars(all=TRUE))
```

eFigure 2 – Model diagnostics, including posteriors for intercept and interactions, as well as chains, for 28-day mortality endpoint.

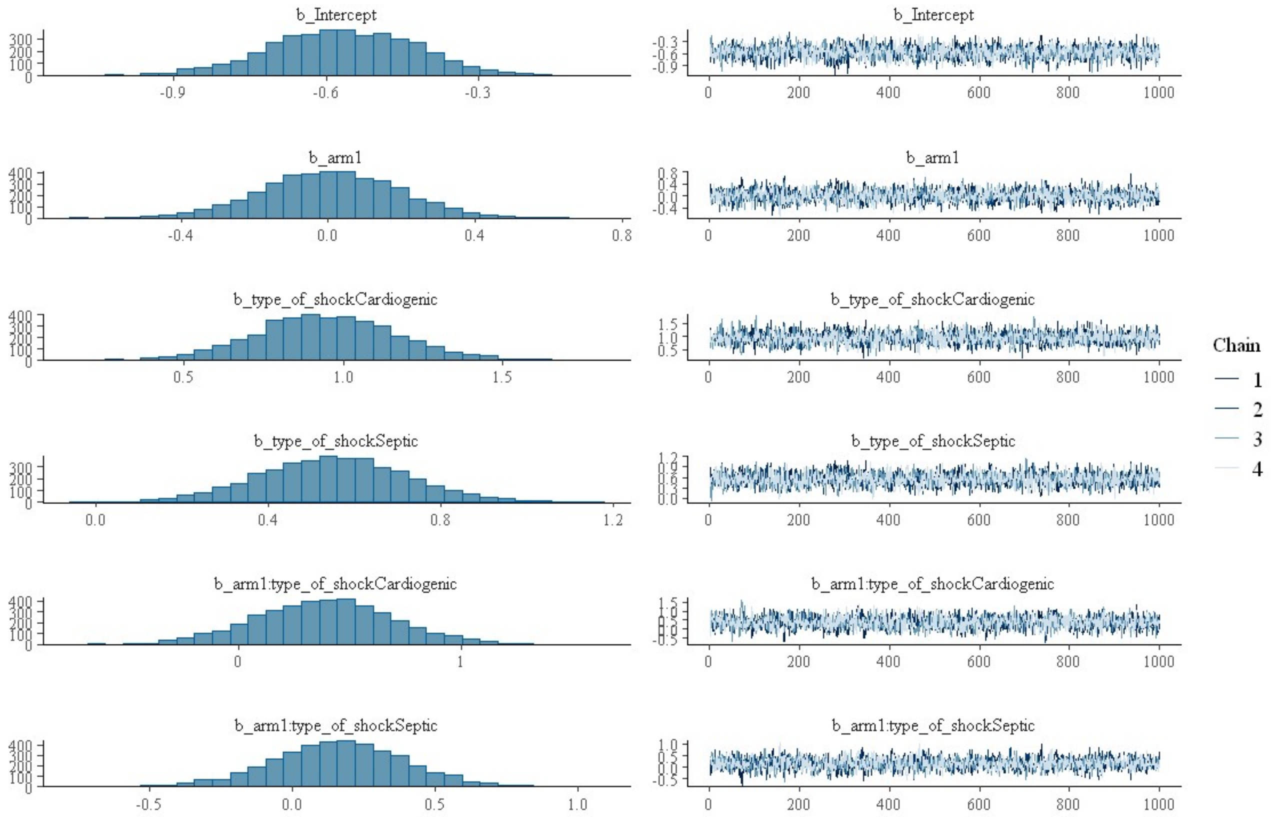

b. Model Syntax for composite endpoint: Note that the neutral prior was applied to intervention arm, but the remaining priors were kept as uninformative (flat). The prior concentrates 95% of its probability mass between odds ratio of 0.5 to 2.0.

```
myprior <- prior(normal(0,0.355), class="b", coef="arm1")
b1 <- brm(composite ~ arm * type_of_shock, family="bernoulli", chains = 4, cores = 4, seed = 123,
prior = myprior, data = df,save_pars = save_pars(all=TRUE))
```

eFigure 3 – Model diagnostics, including posteriors for intercept and interactions, as well as chains for composite endpoints

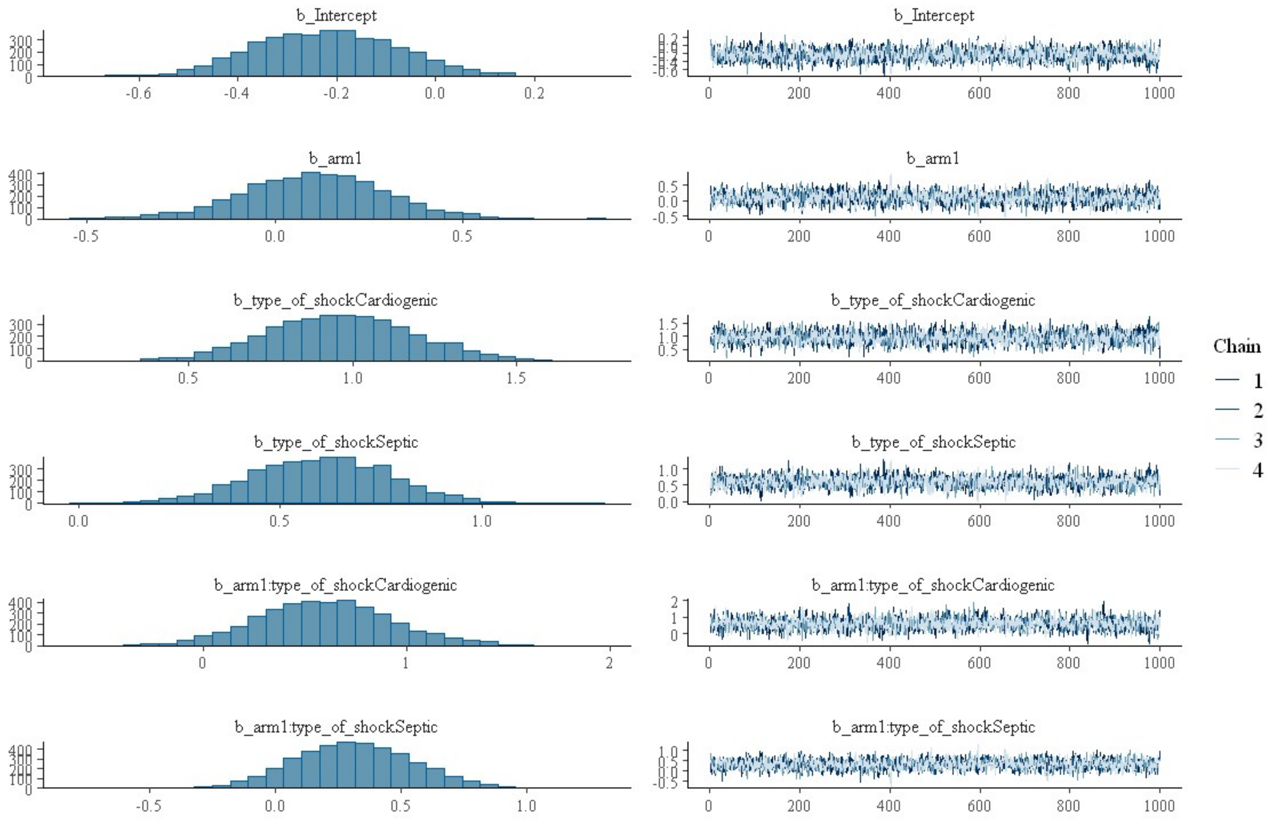

c. Model Syntax for DAFICU28: Note that the neutral prior was applied to intervention arm, but the remaining priors were kept as uninformative (flat). The prior concentrates 95% of its probability mass between an *estimate* of -0.7 to 0.7 days alive.

```
myprior <- prior(normal(0,0.355), class="b", coef="arm1")
b1 <- brm(daficu28 ~ arm * type_of_shock, family="gaussian", chains = 4, cores = 4, seed = 123,
prior = myprior, data = df,save_pars = save_pars(all=TRUE))
```

eFigure 4 – Model diagnostics, including posteriors for intercept and interactions, as well as chains, for DAFICU28.

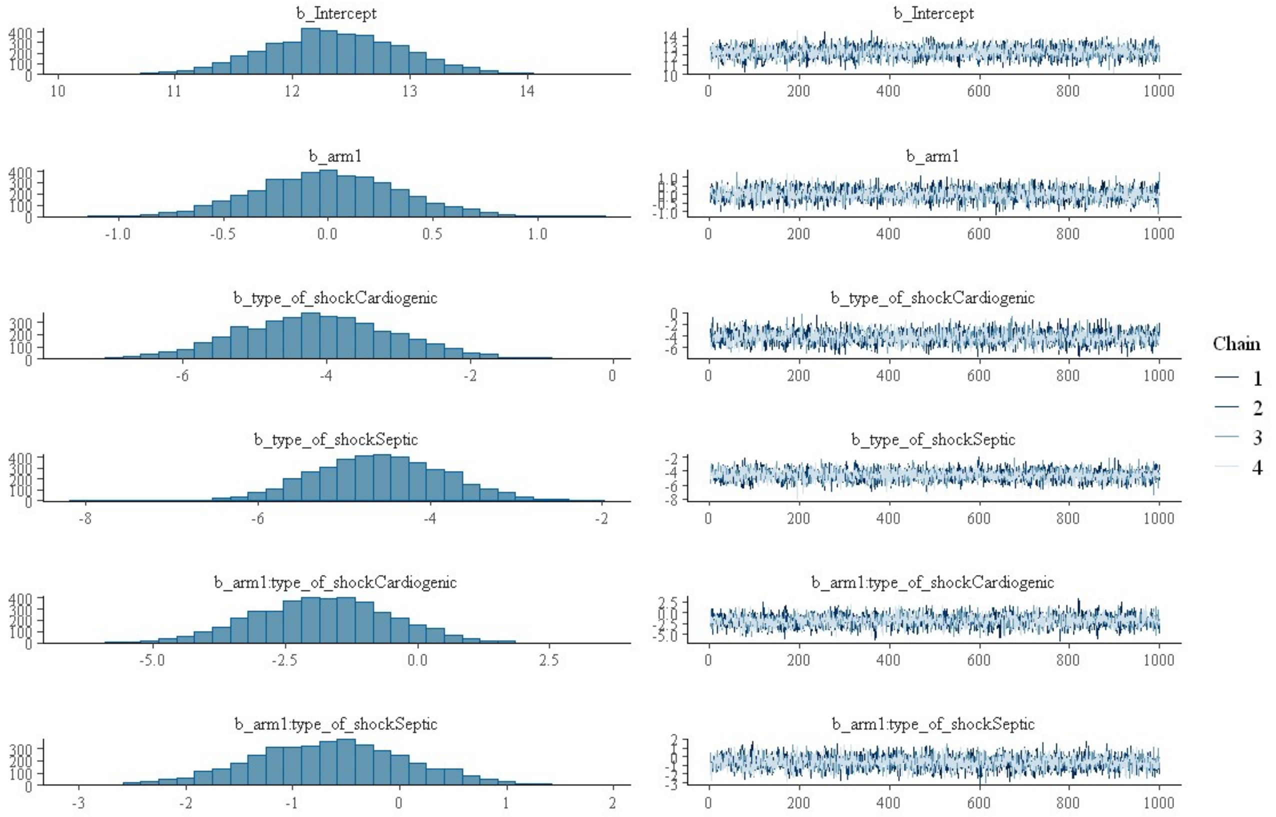

2. APACHE II Customization Results

Customization was necessary due to the lack of crude data on reason for admission allowing original APACHE II predictions to be calculated.

eFigure 5 – APACHE II discrimination (left) and calibration (right) in the SOAP II. Note that AUC for APACHE II was reasonable (0.68) but calibration was excellent with the predicted probabilities never exceeding the observed probabilities beyond 80% confidence levels.

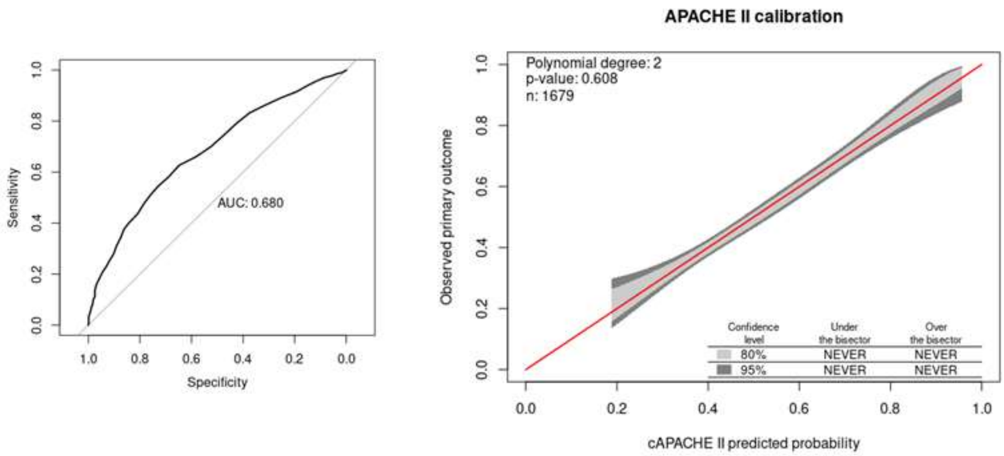

3. Risk-based HTE analysis

Like the Bayesian analysis presented in 2, a similar syntax was used for most models for the Bayesian risk-based HTE analysis. Similarly, for all models Bayes Factors for model with and without interaction were done by creating a model with the interaction and one without it, and then comparing models using brms::bayes_factor(b1,b1ni), where "ni" refers to the model built without interaction. Coefficients and contrasts were extracted using {marginaleffects}

The skeleton syntax for those models were:

```
myprior <- prior(normal(0,0.355), class="b", coef="arm1")
bn <- brm(endpoint ~ arm * type_of_shock, family="family", chains = 4, cores = 4, seed = 123, prior
= myprior, data = df,save_pars = save_pars(all=TRUE))
```

Where endpoint could be death or composite endpoint (using family = "bernoulli") or DAFICU28 (in which case family would be = "gaussian").

Model diagnostic plots are also provided below.

eFigure 6 – Model diagnostics, including posteriors for intercept and interactions, as well as chains, for mortality for the HTE analysis with APACHE II quartiles.

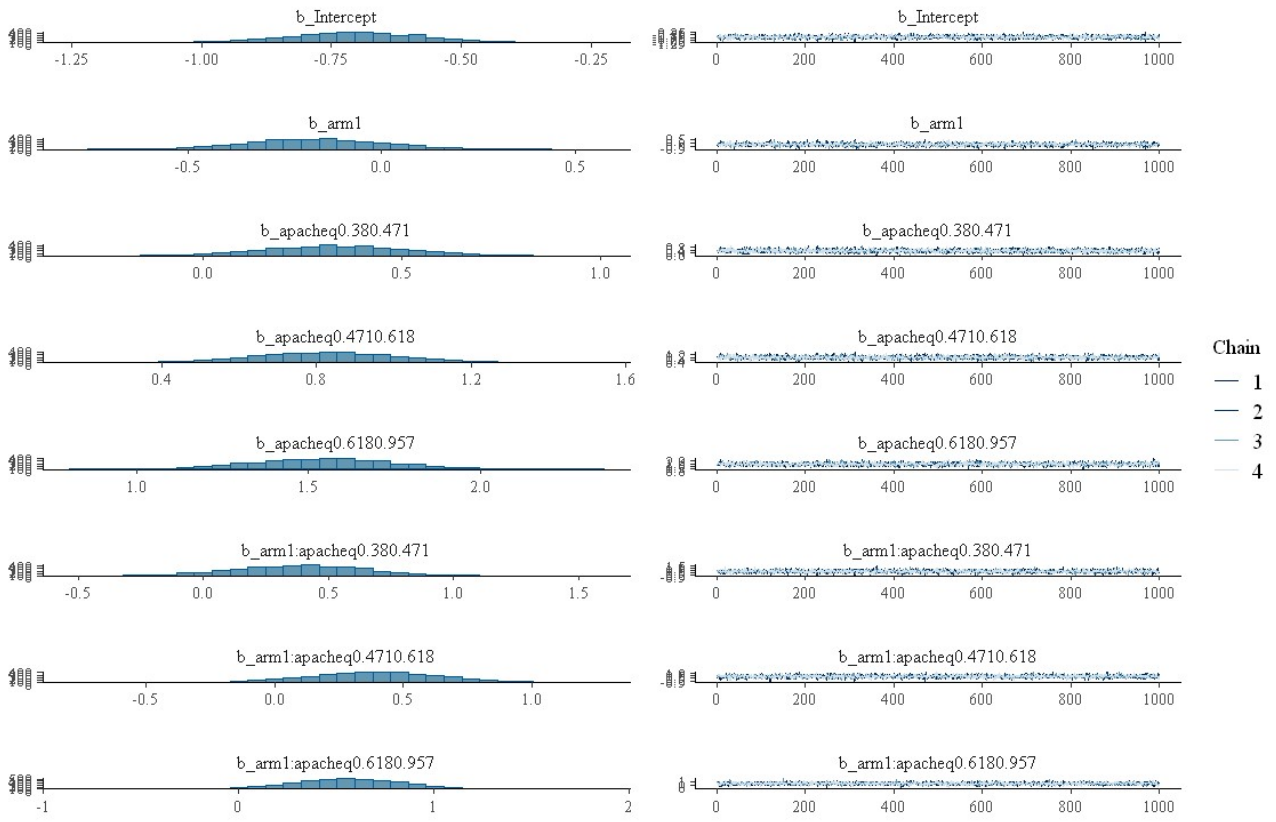

eFigure 7 – Model diagnostics, including posteriors for intercept and interactions, as well as chains, for composite endpoint for the HTE analysis with APACHE II quartiles.

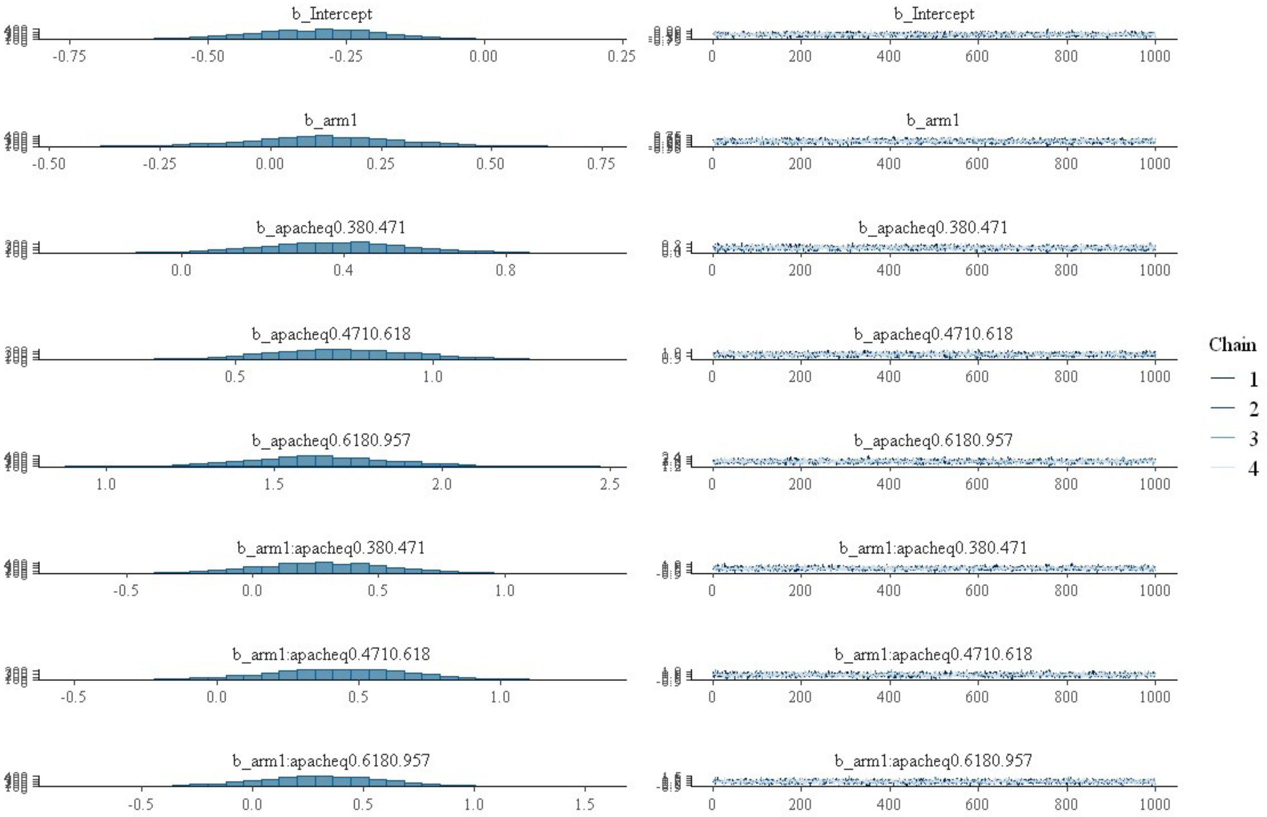

eFigure 8 – Model diagnostics, including posteriors for intercept and interactions, as well as chains, for DAFICU28 endpoint for the HTE analysis with APACHE II quartiles.

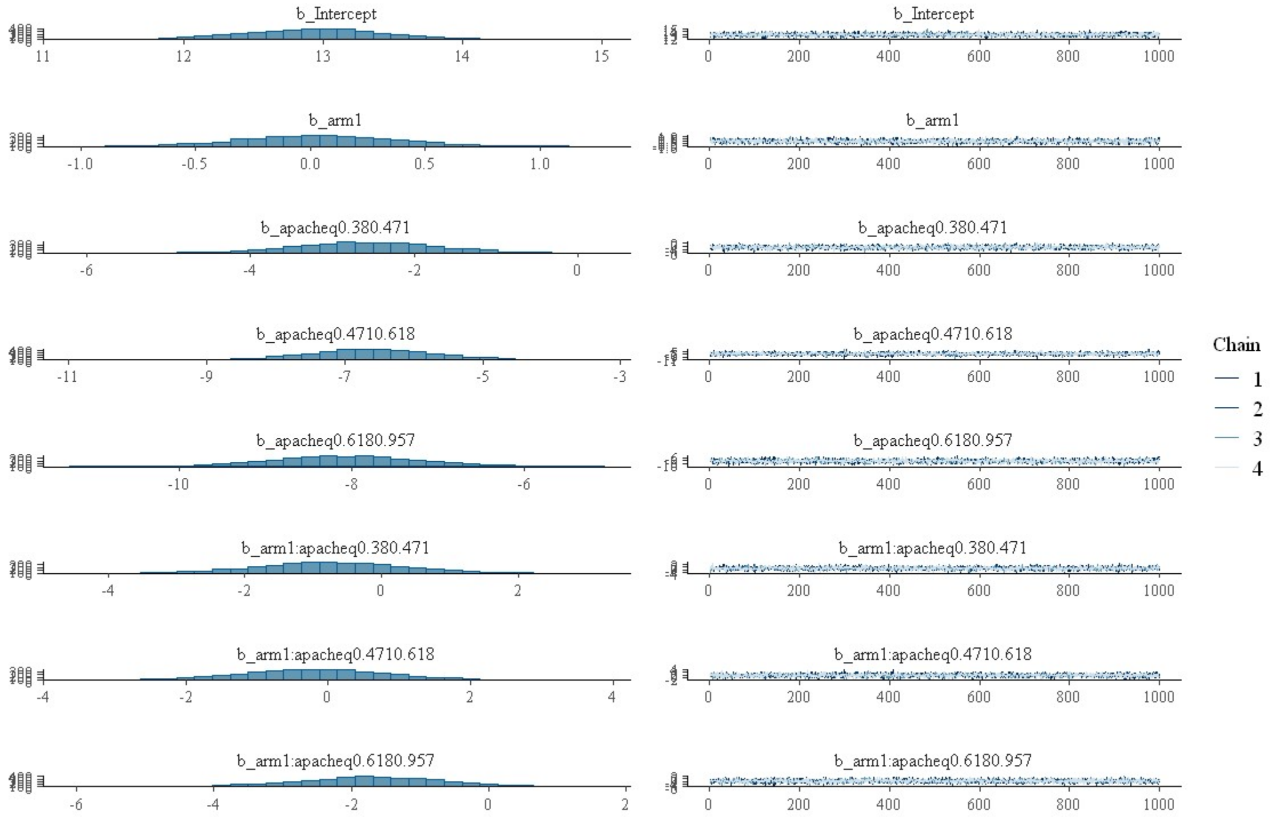

4. Effect-based HTE analysis

This analysis was based on training a model in a train dataset (random 1,010 patients in SOAP II) using a statistical model and using the model in the test dataset (remaining patients) using the composite endpoint as target. The model was adjusted to age, type of shock, cardiomyopathy, and SOFA, all interacting with study arm. Note that priors for effect size for all estimates (not only intervention) were the neutral prior previously defined but that in this situation no flat priors were used in the analysis to make the model further skeptical to extreme effect sizes.

The model trained on train dataset was:

```
myprior2 <- prior(normal(0,0.355),class="b")
bslearner <- brm(composite ~ (age + type_of_shock + cardiomyopathy + sofa)*arm,
family="bernoulli", chains = 4, cores = 4, seed = 123,prior=myprior2, data = train,
save_pars = save_pars(all=TRUE))
```

Once the model was defined, counterfactual probabilities were created in the test set (dftlearner), by changing patients from control to intervention group. This was used to create a summary (s1, below) that included a recommendation for each patient:

```
pred_norepi <- dftlearner %>% mutate(arm = 0) %>% add_epred_draws(bslearner,seed=123) %>%
ungroup() %>% dplyr::select(id,pred_norepi = .epred)
pred_dopamine <- dftlearner %>% mutate(arm = 1) %>%
add_epred_draws(bslearner,seed=123) %>% ungroup() %>% dplyr::select(id,pred_dopamine
= .epred)
preds_all <- bind_cols(pred_norepi,pred_dopamine)
preds_all$cate <- preds_all$pred_dopamine - preds_all$pred_norepi
preds_all$id<-preds_all$id...1
preds_all$id...1<-NULL
preds_all$id...3<-NULL
s1<- preds_all %>% group_by(id) %>%
        summarise(cateavg=median(cate),
        catelow = quantile(cate,probs = 0.025),
        catehigh = quantile(cate,probs = 0.975),
        recommendation = as.factor(ifelse( (sum(cate<0)/n()) > 0.90, "dopamine",
        ifelse( (sum(cate>0)/n()) > 0.90, "norepinephrine","none")))) %>%
        ungroup()
```

The "recommendation" variable is a factor of the recommendation made by the bslearner model to the test set. We then proceed to assess whether receiving an intervention aligned with the recommendation is associated with differences in composite endpoints.

```
ttt<-left_join(dftlearner,s1)
ttt_model <- brm(composite ~ arm * recommendation, family="bernoulli", chains = 4, cores
= 4, seed = 123, prior = myprior, data = ttt,save_pars = save_pars(all=TRUE))
```

Note that the prior here was only applied to the enrolling arm. The model never recommended dopamine, so the diagnostic plots only include the recommendation = "none" (considering recommendation = norepinephrine as default).

eFigure 9 – Model diagnostics, including posteriors for intercept and interactions, as well as chains, for composite endpoint, arm, and recommendation in the test dataset.

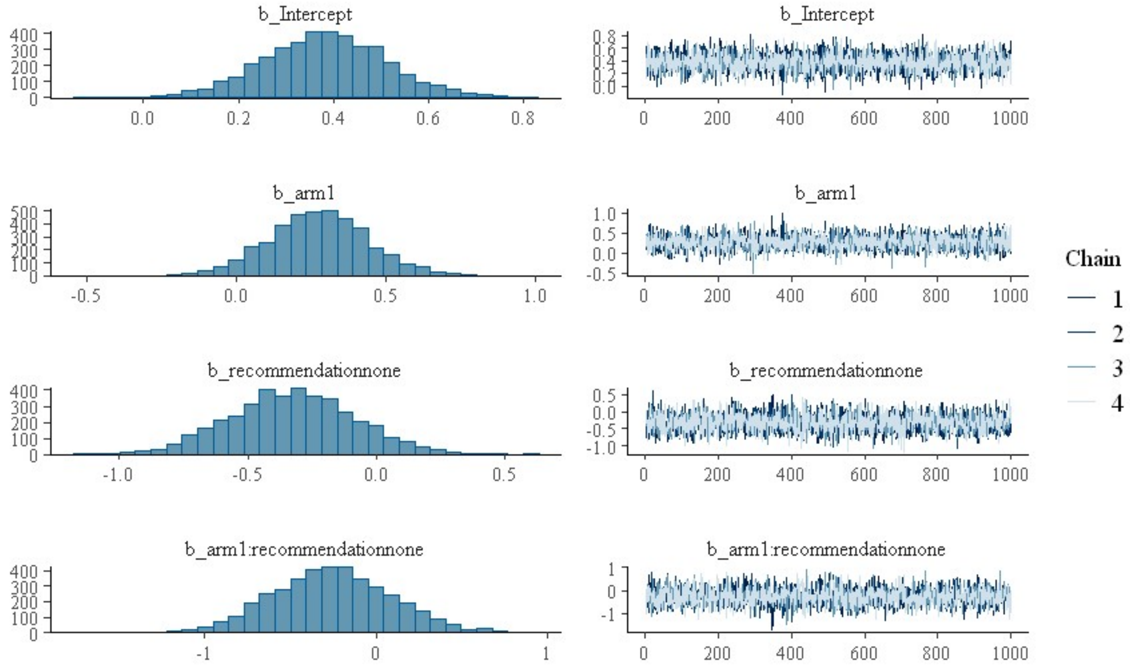

eTable 2 – Comparison of patients in the test set according to S-learner model recommendation.

Characteristic	Norepinephrine<br>N = 858	None<br>N = 821
**ADMISSION**		
**Age, mean (SD)**	64 (14)	70 (12)
**Sex, n (%)**		
Female	213 (44%)	83 (46%)
Male	276 (56%)	97 (54%)
**APACHE II, median (IQR)**	22 (17, 30)	17 (13, 21)
**Cardiomyopathy, n (%)**		
No	285 (58%)	54 (30%)
Yes	204 (42%)	126 (70%)
**Type of shock, n (%)**		
Cardiogenic	64 (13%)	51 (28%)
Other	43 (8.8%)	106 (59%)
Septic	382 (78%)	23 (13%)
**Mechanical Ventilation, n (%)**	363 (74%)	105 (78%)
**Renal replacement therapy, n (%)**	41 (8.4%)	7 (3.9%)
**SOFA, points, mean (SD)**	9.9 (3.3)	5.7 (3.0)
**OUTCOMES**		
**New use of renal replacement therapy, n (%)**	56 (11%)	9 (5%)
**Arrhythmia**	89 (18%)	29 (16%)
**Days Alive and Free of ICU, mean (SD)**	8 (10)	12 (12)
**28-day mortality**	244 (50%)	78 (43%)
**Composite endpoint**	307 (63%)	93 (52%)

5. Other Analysis

eFigure 10 – Probability of death according to heart rate and intervention arm. A spline was added for the hear rate to account for non-linearities. P value for interaction = 0.187.

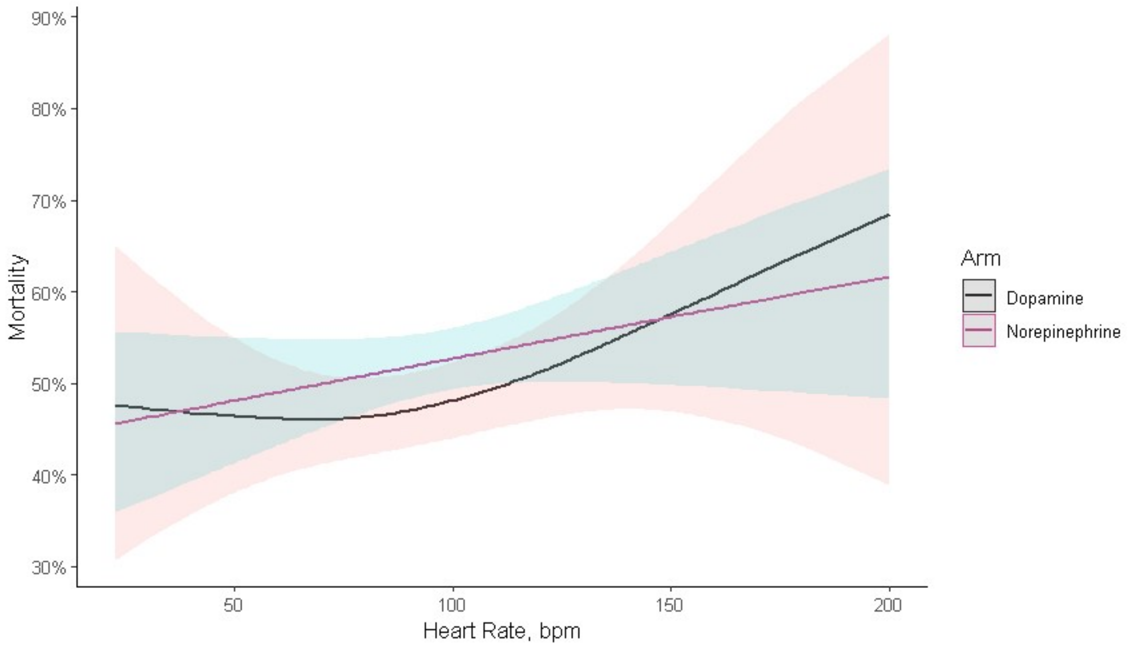

eFigure 11 – Probability of death according to heart rate (in quartiles), type of shock, and intervention arm. P value for interaction between heart rate and arm = 0.48.

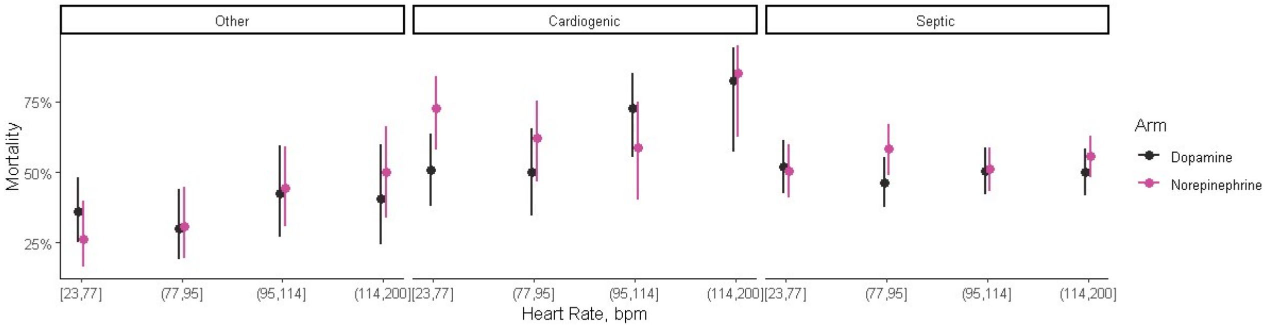

Supplement: Supplementary file 1 — Supplementary Material 1. [file 13054_2024_5016_MOESM1_ESM.pdf]
